# Diversity, Composition, and Specificity of the *Philaenus spumarius* Bacteriome

**DOI:** 10.3390/microorganisms12020298

**Published:** 2024-01-30

**Authors:** Cristina Cameirão, Daniela Costa, José Rufino, José Alberto Pereira, Teresa Lino-Neto, Paula Baptista

**Affiliations:** 1 Centro de Investigação de Montanha (CIMO), Instituto Politécnico de Bragança, Campus de Santa Apolónia, 5300-253 Bragança, Portugal; ccameirao@ipb.pt (C.C.); jpereira@ipb.pt (J.A.P.); 2 Laboratório para a Sustentabilidade e Tecnologia em Regiões de Montanha, Instituto Politécnico de Bragança, Campus de Santa Apolónia, 5300-253 Bragança, Portugal; rufino@ipb.pt; 3 Centre of Molecular and Environmental Biology (CBMA), Department of Biology, University of Minho, Campus de Gualtar, 4710-057 Braga, Portugal; daniela.ffc22@gmail.com (D.C.); tlneto@bio.uminho.pt (T.L.-N.); 4 Research Centre in Digitalization and Intelligent Robotics (CeDRI), Instituto Politécnico de Bragança, Campus de Santa Apolónia, 5300-253 Bragança, Portugal

**Keywords:** meadow spittlebug, endosymbiont, bacteria, metabarcoding

## Abstract

*Philaenus spumarius* (Linnaeus, 1758) (Hemiptera, Aphrophoridae) was recently classified as a pest due to its ability to act as a vector of the phytopathogen *Xylella fastidiosa*. This insect has been reported to harbour several symbiotic bacteria that play essential roles in *P. spumarius* health and fitness. However, the factors driving bacterial assemblages remain largely unexplored. Here, the bacteriome associated with different organs (head, abdomen, and genitalia) of males and females of *P. spumarius* was characterized using culturally dependent and independent methods and compared in terms of diversity and composition. The bacteriome of *P. spumarius* is enriched in Proteobacteria, Bacteroidota, and Actinobacteria phyla, as well as in *Candidatus* Sulcia and *Cutibacterium* genera. The most frequent isolates were *Curtobacterium*, *Pseudomonas*, and *Rhizobiaceae* sp.1. Males display a more diverse bacterial community than females, but no differences in diversity were found in distinct organs. However, the organ shapes the bacteriome structure more than sex, with the Microbacteriaceae family revealing a high level of organ specificity and the Blattabacteriaceae family showing a high level of sex specificity. Several symbiotic bacterial genera were identified in *P. spumarius* for the first time, including *Rhodococcus*, *Citrobacter*, *Halomonas*, *Streptomyces*, and *Providencia*. Differences in the bacterial composition within *P. spumarius* organs and sexes suggest an adaptation of bacteria to particular insect tissues, potentially shaped by their significance in the life and overall fitness of *P. spumarius*. Although more research on the bacteria of *P. spumarius* interactions is needed, such knowledge could help to develop specific bacterial-based insect management strategies.

## 1. Introduction

Insects are invertebrates that intimately depend on their microbiota for vital functions, particularly on endosymbionts. These microorganisms, living inside insects, provide fitness advantages to the insect, with variable effects according to the colonized organ. For example, the gut microbiota is vital for insect adaptation to nutrient-limited environments by providing essential amino acids that cannot be obtained through feeding [1]. On the other hand, the endosymbionts present in the host reproductive organs can manipulate insect reproduction through cytoplasmatic incompatibility, male killing, feminization, or parthenogenesis [2]. In these processes, the production of female offspring is increased as the endosymbiont selectively eradicates the members of their host population that cannot transmit the infection, thus benefiting the maternal inheritance of the endosymbiont [2]. While distinct functions have been reported for endosymbionts in different host organs, the factors determining their assemblage are still not fully understood. Both host species and diet appear to be the most relevant drivers of insect microbiota, but tissue type, life stage, and sample origin or habitat may also have some impact [3,4]. Although sex has been less frequently considered in studies of insect microbial communities, there is increasing evidence that sex can affect the structure of these communities [5,6]. Compared to other insect species, knowledge of the factors shaping microbial assemblages is particularly limited in *Philaenus spumarius* L. (Hemiptera: Aphrophoridae). This xylem sap-feeding insect has recently garnered scientific attention due to its capacity to transmit important plant pathogens, such as *Xylella fastidiosa* [7]. In Europe, *X. fastidiosa* has been causing a significant economic impact, primarily in olive groves, following outbreaks of the Olive Quick Decline Syndrome (OQDS) caused by this bacterium [8]. *Philaenus spumarius* has been recognized to play a crucial role in disseminating this bacterium, particularly in European regions heavily impacted by the OQDS epidemic [9]. The microbial community associated with *P. spumarius* has predominately been assessed using a PCR-specific primer-based approach, primarily targeting the detection of specific endosymbiont genera, notably *Wolbachia* [10,11], as well as *Arsenophonus*, *Hamiltonella*, *Cardinium,* and *Rickettsia* [12]. Only recently, the bacterial community associated with *P. spumarius*, as well as five other species of the genus *Philaenus*, was comprehensively examined using metabarcoding techniques designed to capture multiple taxa [13]. Examining the complete bacteriome rather than focusing on a single bacterial species is expected to offer a more comprehensive understanding of bacterial communities and their functions within *P. spumarius*. This approach not only leads to the discovery of previously unknown species but also allows for exploring competitive and cooperative interactions among bacterial species [14], which can have significant functional implications in *P. spumarius*. Furthermore, the influence of both the host’s organ and sex on the composition of the entire bacterial community associated with *P. spumarius* has never been investigated. A thorough understanding of how these microbial communities assemble and function can reveal whether specific benefits provided by endosymbionts are contingent upon the sex or organ of the host. Indeed, identifying bacterial members specific to a particular sex or organ of *P. spumarius* may provide new insights into their potential impacts on host fitness. This “specificity” is a concept often used in symbiotic interactions and has been considered an important determinant for the host–microbial interaction outcome [15]. Such knowledge may have ecological and practical implications, including their potential for controlling *P. spumarius* via the exploitation and manipulation of the associated endosymbionts.

Therefore, in this work, the bacterial community present in different organs (abdomen, head, and genitalia) of both males and females of *P. spumarius* was studied by metabarcoding and cultural approaches. The inclusion of both approaches not only provides complementary information but also opens the door for utilizing cultivable bacteria in strategies aimed at controlling *P. spumarius*. Specifically, this research seeks to address the following questions: (i) How do bacterial communities differ in terms of diversity and composition with *P. spumarius* sex (males and females) and organ (abdomen, head, and genitalia)? (ii) Can host sex and organs influence the composition of associated bacterial communities? (iii) Can we identify specific communities associated with each insect sex and organ?

## 2. Materials and Methods

### 2.1. Philaenus spumarius Collection and Processing

Adults of *P. spumarius* were collected from natural ground vegetation in four locations in northern Portugal between September and November 2019 (see Table S1). The collection was conducted using an entomological sweep net with a diameter of 38 cm, and no particular plant hosts were considered during sweeping. In total, 91 adult specimens were collected, comprising 33 males and 58 females. Upon collection, the insects were carefully transferred to a plastic bag, and euthanasia was carried out by injecting ether. Subsequently, they were transported in a cooler box to the laboratory and immediately identified and separated by sex under a binocular stereomicroscope (Leica EZ4), according to the morphological descriptions provided by Germain et al. (2020) [16]. The insects were then subjected to a sterilization process previously optimized by us. In particular, the efficiency of sodium hypochlorite and ethanol when used alone or in combination, their concentration, and the distinct periods of *P. spumarius* adults’ exposure were tested. The optimized protocol included sequential immersion of adults in 70% (*v*/*v*) ethanol for 1 min, followed by 3% (*v*/*v*) sodium hypochlorite for 1 min. This was followed by three rinses, each lasting 1 min, with sterile distilled water. The effectiveness of sterilization was confirmed by imprinting the insect surface onto Plate Count Agar (PCA, HiMedia, Mumbai, India) culture medium. After surface sterilization, the insects were dissected under aseptic conditions using a binocular stereomicroscope (Leica EZ4). During the dissection, each adult was partitioned into distinct parts, including the head, abdomen, genitalia, wings, and legs. The head, abdomen, and genitalia were further used for bacterial assessment, while the wings and legs were discarded.

### 2.2. Bacterial Isolation and Molecular Identification

A total of 8 males and 33 females of *P. spumarius* were utilized to isolate bacteria from the various organs. Three distinct culture media were used for bacterial isolation, which was prepared by using plate count agar (PCA) as a basal medium because it supports the growth of a broad spectrum of bacteria. The culture media used included PCA (HiMedia, Mumbai, India), PCA supplemented with 0.5% (*w*/*v*) crushed insects (a mixture of insects retained in the sweeping net during *P. spumarius* collection), or 0.5% (*v*/*v*) of an insect medium supplement (Sigma-Aldrich, St. Louis, MO, USA), frequently used for insect cell culture. These two latter media were used to replicate the nutrient availability of the insect host since most endosymbionts have specific nutritional needs and cannot grow in simpler media [17]. For the isolation process, each organ was individually ground in 90 µL of phosphate-buffered saline with Tween 80 (PBST: 137 mM NaCl, 2.7 mM KCl, 10 mM Na_2_HPO_4_, 1.8 mM KH_2_PO_4_, 0.01% Tween 80), using a disposable pestle in a 1.5 mL Eppendorf tube. The obtained macerate (10 µL) was plated in each culture medium. These plates were subsequently incubated at room temperature (22–24 °C) and daily checked for bacterial growth. Single colonies of each bacterial morphotype were counted, and their abundance was expressed in Log Colony-Forming Units (CFU) per mL. To obtain a pure culture, one isolate from each bacterial morphotype was sub-cultured using the same medium used for its isolation. Pure isolates were further identified by sequencing the V1-V3 region of the 16S rRNA gene. Genomic DNA was extracted using the REDExtract-N-AmpTM Plant PCR kit from Sigma-Aldrich, St. Louis, MO, USA. For barcode amplification, 2 µL of extracted DNA was combined in a 20 µL PCR mixture containing 1x reaction buffer (BIORON GmbH, Römerberg, Germany), 2.5 mM MgCl_2_, 200 mM dNTPs, 0.2 µM primer 27F (5′-AGAGTTTGATCCTGGCTCAG-3′) [18], 0.2 µM primer 534R (5′-ATTACCGCGGCTGCTGG-3′) [19], and 1.25 U DFS-Taq DNA Polymerase (BIORON GmbH, Römerberg, Germany). The PCR procedure was performed in a MyCyclerTM Thermocycler (Bio-Rad, Hercules, CA, USA) using the following program: 94 °C for 5 min, followed by 25 cycles of 94 °C for 40 s, 54 °C for 50 s, and 72 °C for 30 s, and a final extension of 72 °C for 7 min. The amplified region was sequenced by Macrogen Inc. (Madrid, Spain), and taxonomic classification was performed using the NCBI database and the BLAST algorithm [20]. Classification into operational taxonomic units (OTUs) was based on the criteria of the lowest e-value, a higher percentage of identity (>98% for species level; 95% to 97% for genus level), and query coverage. The obtained sequences were submitted to the NCBI database with accession numbers OM243853 to OM243910. Detailed information regarding the identified bacterial sequences is summarized in Table S2.

Pure cultures of each isolate were preserved in a 30% glycerol (*v*/*v*) solution, stored at −80 °C, and deposited in the CIMO-CC (Mountain Research Center Culture Collection) at the Polytechnic Institute of Bragança (Bragança, Portugal). 

### 2.3. DNA Extraction and 16S Illumina Sequencing

In total, 25 males and 25 females were used to evaluate the bacterial community of their various organs by metabarcoding. For each sex, five replicates, each consisting of five samples, were generated for each organ and used for DNA extraction. The DNA extraction was performed after organ homogenization with liquid nitrogen, utilizing the Speedtools Tissue DNA Extraction kit from BIOTOOLS, Madrid, Spain. The quantification of extracted DNA was assessed by spectrometric measurements using the VWR mySPEC microvolume spectrophotometer (VWR, Carnaxide, Portugal). At the same time, the integrity of the DNA was checked by loading 3 µL of DNA into a 0.8% (*w*/*v*) agarose gel. The bacterial communities were assessed through a metabarcoding approach using the Illumina MiSeq platform (Illumina Inc., San Diego, CA, USA) with paired-end sequencing (2 × 250 bp) for the V4 region of the 16S rRNA gene and outsourced to Genoinseq (Cantanhede, Portugal). Briefly, this platform utilizes a sequencing-by-synthesis approach, where fluorescently labeled nucleotides are incorporated into growing DNA strands and the emitted light signals are captured to determine the sequence of the DNA. The sequencing procedure was performed through a nested PCR with the primers 27F: 5′-AGAGTTTGATCMTGGCTCAG-3′ and 1512R: 5′-ACGGCTACCTTGTTACGACTT-3′ [18], followed by a second PCR utilizing the primers 515F: 5′-GTGCCAGCMGCCGCGGTAA-3′ and 806R: 5′-GGACTACHVGGGTWTCTAAT-3′ [21], also adding indexes and sequencing adapters.

### 2.4. 16S Illumina Sequencing Data Processing

Demultiplexed raw reads were initially subjected to trimming with the Sickle tool [22] based on the FastQC [23] quality report and applying the default parameters. After trimming, read errors from sequencing were corrected using the Bayeshammer module from the SPAdes package [24]. The paired-end reads were merged by overlapping regions using USEARCH v11 [25], followed by a quality report with FastQC [23] and filtering parameters determined based on expected amplicon size. The filtering parameters were applied with the ea-utils package [26]. The determination of amplicon sequence variants (ASVs) and their taxonomic classification was performed with MICCA [27], utilizing SILVA release 132 [28] as the reference database. Unclassified ASVs were manually blasted against the NCBI database to confirm their unclassified status. If a new classification was identified, the ASVs were maintained in the dataset. Otherwise, they were discarded. All obtained datasets were then subsampled to the lowest number of reads found in all samples, which are 8601 reads (found in the FA5 sample, a female abdomen sample), by using QIIME 1.9.0 [29], to mitigate biases due to differences in sampling depth. The raw sequence reads and respective metadata were deposited in the NCBI Sequence Reads Archive (SRA) database under the BioProject ID PRJNA797278.

### 2.5. Data Analysis

All data analyses and graphic representations were performed using the R software version 4.0.2 [30]. The diversity, abundance, and composition of bacterial communities inhabiting *P. spumarius* were compared between sexes (male vs. female) and organs (abdomen, head, and genitalia). Diversity was evaluated by calculating the Shannon-Wiener index through the diversity() function of the vegan package [31]. To test for differences between means, data were first subjected to the requirements to perform parametric tests, and in a positive case, a two-way analysis of variance (ANOVA) was performed, followed by a Tukey’s post hoc test. When the data did not meet these requirements, the Kruskal–Wallis non-parametric test was applied, followed by a pairwise Wilcoxon test. All *p*-values under 0.05 were considered statistically significant, and *p*-values from pairwise multiple comparisons were adjusted using the Benjamini and Hochberg correction [32].

For metabarcoding data, a Permutational Multivariate Analysis of Variance (PERMANOVA) was conducted to test whether insect sex and organ could explain the differences in the composition of bacterial communities associated with *P. spumarius*. PERMANOVA and pairwise PERMANOVA were performed using the Bray–Curtis dissimilarities using the adonis() function from the vegan package [31] and pairwise.adonis() function from the pairwise Adonis package, respectively [33]. To visualize differences in community composition, a non-metric multidimensional scaling (NMDS) was conducted using Bray–Curtis dissimilarities with the meta (MDS) function from the vegan package [31]. Kruskal’s stress value was computed to gauge the NMDS ordination model’s adequacy, with a value typically considered acceptable if it falls below 0.2 [34].

A specificity analysis was performed to identify bacterial members associated explicitly with a particular sex (male or female) or organ (abdomen, head, or genitalia) of *P. spumarius*. For this analysis, a family dataset was used (considering only those families detected in at least 10% of samples), and specificity index values (Spec) for each family were determined using the function phy_or_env_spec() of the specificity package [35]. This analysis was plotted as a violin for the organ and sex, in which the total area represented all bacterial families. Compared to other methods, this analysis is not sensitive to occupancy or the number of samples, indicating the extent to which a taxon occupies a narrower range of a variable than expected by chance [35]. The specificity index spans from −1 to 1, where −1 represents perfect specificity, 0 signifies no deviation from the null model, and 1 denotes perfect cosmopolitanism [35].

## 3. Results

### 3.1. Diversity of Bacterial Community Associated with P. spumarius

**Figure 1:**
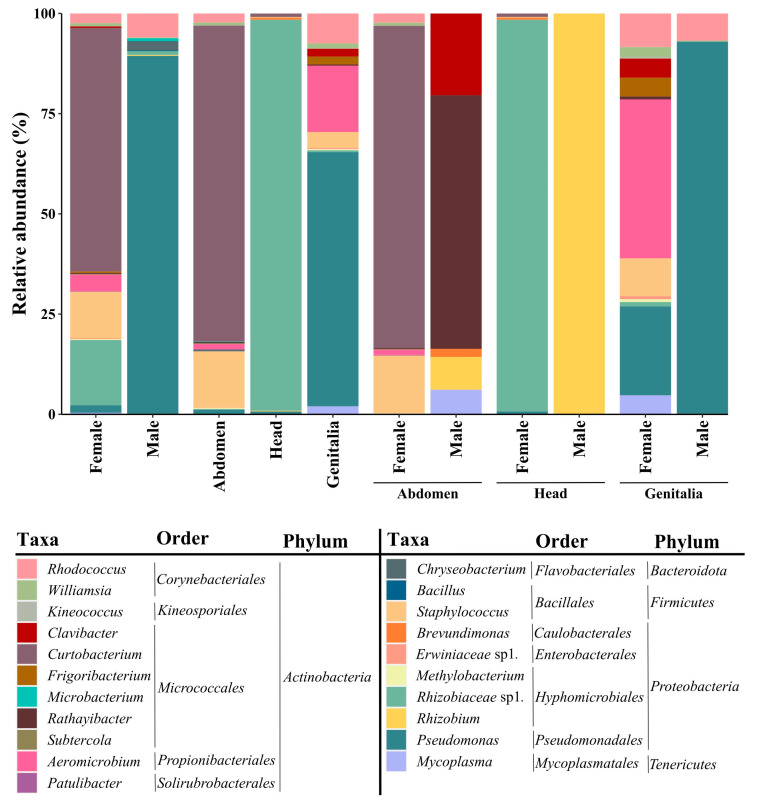
Relative abundance of identified operational taxonomic units (OTUs) from bacterial isolates obtained from females and males of *Philaenus spumarius* in different sexes and organs (abdomen, head, and genitalia) and in organs from each insect sex.

In the culture-dependent approach, no significant differences were found among the three culture media concerning their ability for bacterial growth (*p* = 0.533). Nevertheless, we observed a greater diversity of bacteria with PCA medium (5.84 Log CFU/mL, corresponding to 30 OTUs) compared to PCA supplemented with 0.5% (*w*/*v*) crushed insects (5.80 Log CFU/mL, 26 OTUs) or supplemented with 0.5% (*v*/*v*) insect medium (5.81 Log CFU/mL, 27 OTUs). All cultivated bacterial isolates (6.29 Log CFU/mL) resulted in the identification of 36 OTUs belonging to 19 genera, 14 families, and 5 phyla (Figure S1a). Regarding colonization, females showed a slightly higher frequency of bacterial colonization (54.5% of total female organs) than males (50.0%). The most abundant genus isolated from females was *Curtobacterium* (5.85 Log CFU/mL, accounting for 60.8% of the total isolates). In contrast, males were mostly colonized by members of the *Pseudomonas* genus (5.08 Log CFU/mL, 89.4% of the total isolates) (Figure 1; Table S3). Although no statistically significant differences were observed in the cultivable bacterial diversity (*p* = 0.77) among *P. spumarius* organs, genitalia showed higher colonization frequency (24.4% of the total analyzed genitalia were colonized by bacteria) and richness (23 OTUs) than the abdomen (17.1% and 18 OTUs) and head (12.2% and 6 OTUs). The most abundant bacterial genus in genitalia was *Pseudomonas* (5.14 Log CFU/mL, accounting for 63.4% of the total isolates), while the abdomen was dominated by *Curtobacterium* (5.85 Log CFU/mL, 78.8% of the total isolates) and the head by *Rhizobiaceae* sp1. (5.27 Log CFU/mL, 97.7% of the total isolates) (Figure 1; Table S3). However, the bacterial community composition found in each organ depended on the sex of *P. spumarius* (Figure 1; Table S3). While the *Pseudomonas* genus dominated in males’ genitalia (5.08 Log CFU/mL, corresponding to 93.0% of all male genitalia isolates), females’ genitalia were dominated by *Aeromicrobium* (4.56 Log CFU/mL, 39.6%) and *Pseudomonas* (4.31 Log CFU/mL, 22.2%). Furthermore, heads and abdomens revealed sex-dependent differences. The female head was enriched in *Rhizobiaceae* sp1. (5.27 Log CFU/mL, accounting for 97.7% of female head isolates), and the males were colonized only with *Rhizobium* (2.52 Log CFU/mL). Also, the female’s abdomen was dominated by the genus *Curtobacterium* (5.85 Log CFU/mL, corresponding to 80.3% of all female abdomen isolates), while the males were dominated by *Rathayibacter* (4.01 Log CFU/mL, 63.3%).

In the metabarcoding approach, a total of 702,386 high-quality sequence reads from all 30 samples were identified as belonging to bacteria (Table S4). The number of reads per sample ranged from 8601 to 56,369, averaging 23,412 ± 10,044 reads per sample. To reduce the effects of various sequencing depths, all samples were subsampled to 8601 reads. All datasets resulted in 7774 ASVs belonging to 127 genera, 75 families, 48 orders, 19 classes, and 9 phyla. The majority of ASVs belong to the phylum Proteobacteria (37.1% of classified sequences), followed by Bacteroidota (formally known as Bacteroidetes) (28.1%) and Actinobacteria (23.3%). The most abundant genera were *Candidatus* Sulcia (Bacteroidota, 26.4% of classified sequences) and *Cutibacterium* (Actinobacteria, 15.4%) (Figure S1b). The diversity captured in each sample, assessed using Good’s coverage, exceeded 98.6%, indicating that the depth of sequencing was sufficient for evaluating the diversity of bacterial communities associated with *P. spumarius*. Nevertheless, the metabarcoding was unable to detect some genera (*Clavibacter*, *Kineococcus*, *Mycoplasma*, *Patulibacter*, *Rathayibacter*, *Rhizobium*, and *Subtercola*) obtained via culturing.

**Figure 2:**
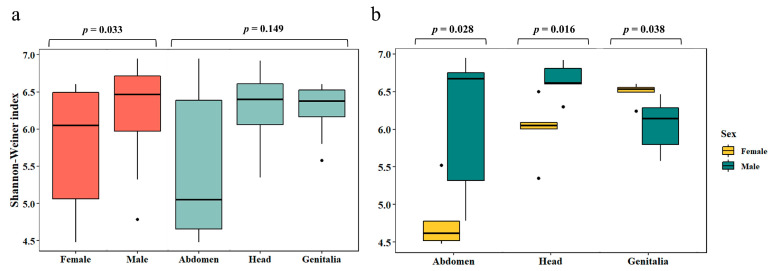
Boxplots showing the diversity (Shannon-Weiner index) of the bacterial community in females and males of *Philaenus spumarius* in different organs (abdomen, head, and genitalia) detected by a culture-independent approach. Results are shown for whole diversity for each sex or organ (**a**) and for diversity comparison among sexes within each organ (**b**). Boxplots depict medians (central horizontal lines), the inter-quartile ranges (boxes), 95% confidence intervals (whiskers), and outliers (black dots). Significant differences between values are represented by horizontal lines.

The Shannon-Weiner index used to estimate bacterial diversity did not differ significantly among *P. spumarius* organs (*p* = 0.149). However, differences in Shannon–Weiner index were observed between females and males, with males displaying a significantly higher diversity (up to 1.1-fold, *p* = 0.033) than females (Figure 2a). These differences were mainly noticed within the communities inhabiting both the abdomen and head of *P. spumarius* (Figure 2b). In genitalia, a different pattern was observed, with significantly higher diversity in females (up to 1.1-fold; *p* = 0.038) compared to males. When analyzing each sex separately, females displayed significantly higher bacterial diversity in the head and genitalia (up to 1.4-fold, *p* = 0.024) when compared to the abdomen. Additionally, there was a significant difference in diversity between the female genitalia and head (*p* = 0.032), with the first being more diverse. In contrast, males showed a similar bacterial diversity among the three organs surveyed (*p* = 0.273).

**Figure 3:**
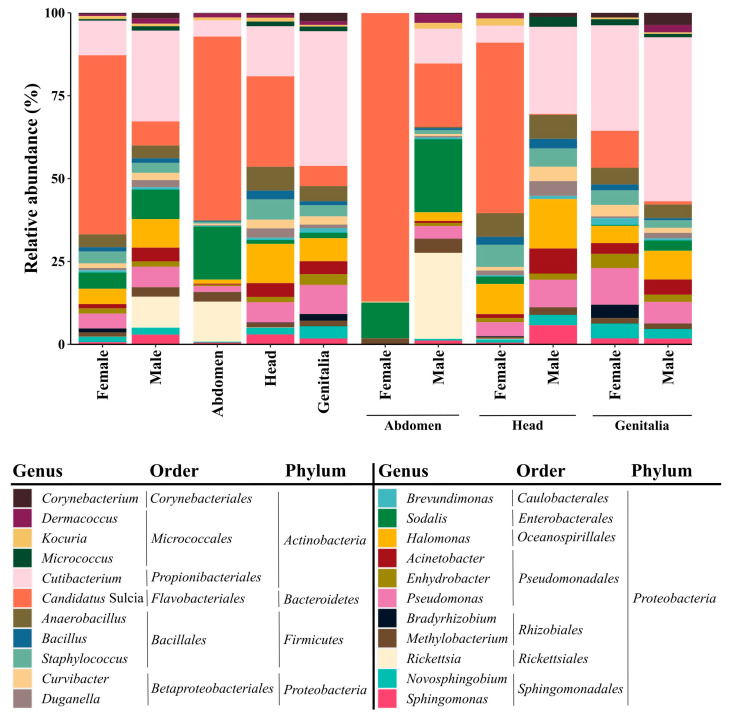
Relative abundance of bacterial ASVs in females and males of *Philaenus spumarius*, in different organs (abdomen, head, and genitalia), and in the different organs of both insect sexes. The results are sorted by genus, and the corresponding orders and phyla are indicated. Only genera representing more than 1% of the relative abundance of total reads in the complete dataset are represented.

Males were dominated by the genus *Cutibacterium* (27.4% of the total abundance found in this sex), whereas females displayed a high abundance of bacteria of the *Candidatus* Sulcia genus (54.0%) (Figure 3). Regarding the bacteria found in different organs, and irrespective of the sex, the bacterial community of the abdomen and head was dominated by the genus *Candidatus* Sulcia (55.4% of the total abundance in the abdomen and 27.3% in the head), while the genitalia’s bacterial community was dominated by *Cutibacterium* (40.6%) (Figure 3). The bacterial communities in different organs varied depending on sex (Figure 3). While the female abdomen was dominated by the genus *Candidatus* Sulcia (87.0%), the male abdomen was dominated by the genus *Rickettsia* (26.0%) and *Sodalis* (22.0%). Indeed, *Rickettsia* was almost exclusively found in the male abdomen. Also, the female head was dominated by the genus *Candidatus* Sulcia (51.4%). In contrast, the male head was dominated by *Cutibacterium* (26.2%) (Figure 3). Both female and male genitalia were characterized by the high abundance of the *Cutibacterium* genus (31.8% in females and 49.5% in males).

When considering the bacterial community reported to establish symbiotic interactions with insect hosts, we found a high diversity of bacteria, detecting 12 symbiotic genera in *P. spumarius* (Figure S2). Among these, *Candidatus* Sulcia, *Halomonas*, and *Sodalis* were symbiotic genera found in all organs of both males and females. Other bacteria genera were exclusive to the genitalia (*Citrobacter*, *Flavobacterium*, and *Rhodococcus*), to the females (*Flavobacterium*), or to the males (*Citrobacter* and *Rhodococcus*). Also, Streptomyces was only found in female heads.

### 3.2. Factors Affecting the Shaping of Bacterial Community Composition in P. spumarius

**Figure 4:**
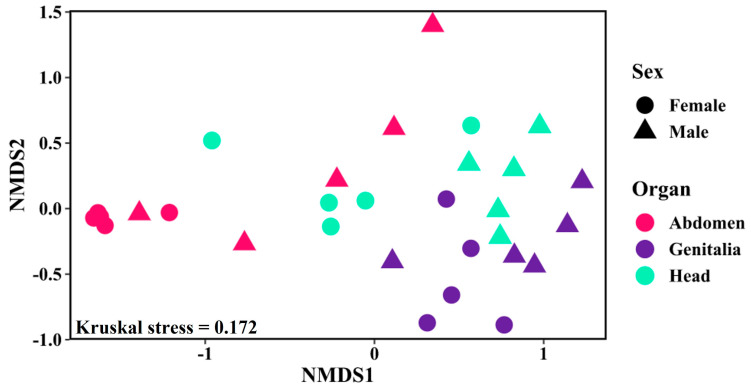
Non-metric multidimensional scaling (NMDS) plot clustering the bacterial community associated with *Philaenus spumarius* in each sex (female and male) and organ (abdomen, head, and genitalia). The Bray–Curtis coefficient was used as a measure of similarity between bacterial populations. The Kruskal’s stress value is also shown (values less than 0.2 represent good ordination plots).

The bacterial communities associated with *P. spumarius* were compared among sexes and organs, considering only the metabarcoding results. Indeed, this approach proved to be more effective in capturing the diversity of entire bacterial communities, including both cultured and uncultured bacteria. The PERMANOVA analysis based on the Bray–Curtis index showed that mostly the “organ” but also the “sex” factors contributed significantly (*p* = 0.001) to the shaping of whole bacterial communities, explaining about 26% and 14% of bacterial composition variation, respectively (Table S5). The NMDS plot corroborates these results by clustering together the samples according to “organ” or “sex” (Figure 4). Furthermore, the bacterial communities were significantly separated by organs (R^2^ = 0.260, *p* = 0.001), with particularly distinct differences observed between the abdomen and genitalia (R^2^ = 0.296, *p* = 0.001) (Table S5). Such distinction was less evident when considering head and genitalia (R^2^ = 0.165, *p* = 0.001) or abdomen and head (R^2^ = 0.155, *p* = 0.015). These differences may be due to the exclusive occurrence of many bacterial members in each organ, as described previously. Although less significant (R^2^ = 0.136, *p* = 0.001), the females and males also harbored different bacterial communities. This dissimilarity was greater when considering the abdomen (R^2^ = 0.373, *p* = 0.016) and head (R^2^ = 0.331, *p* = 0.016) than in the case of the genitalia (R^2^ = 0.160, *p* = 0.016) (Figure 4; Table S5). 

### 3.3. Specificity of Bacteria for a Particular Sex or Organ of P. spumarius

**Figure 5:**
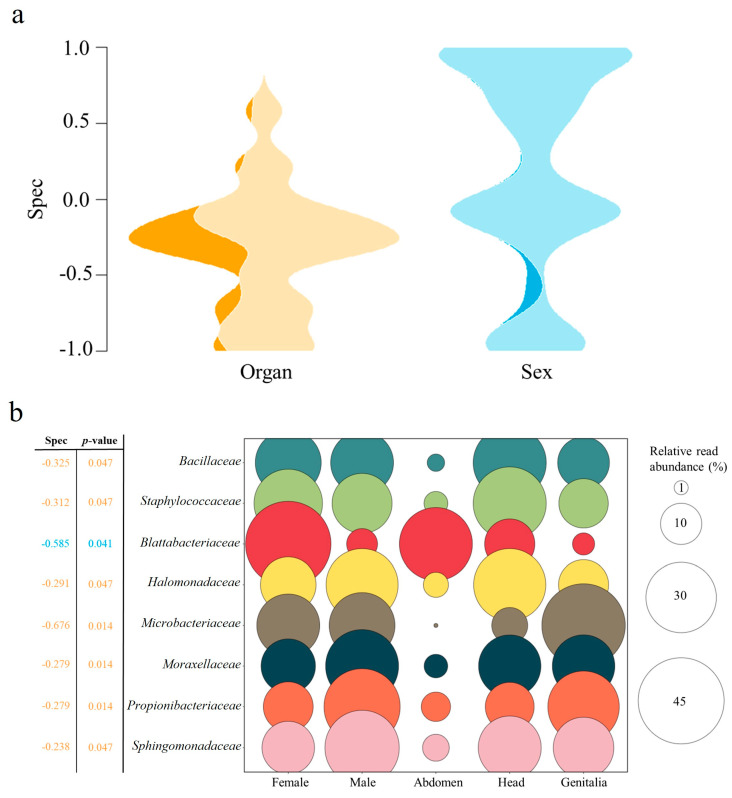
Specificity of the bacterial families for the organ (abdomen, head, and genitalia) and sex (female and male) of *Philaenus spumarius*. The specificity index values (Spec) for 41 families are plotted as violins for organ and sex, and those families with a statistically significant specificity index are represented with a dark color (**a**). The relative read abundance of detected specific families is plotted considering either the organ or the sex of *P. spumarius* (**b**). Spec and *p*-values in orange are particular to the organ, and in blue, they are specific to the sex.

Our results show that the “organ” and, to a lesser extent, the “sex” factor have a pronounced effect on the bacterial composition associated with *P. spumarius*. Therefore, a specificity analysis was conducted to identify those bacterial members that exhibit specificity and a preference for a particular sex (male vs. female) or organ (abdomen, head, or genitalia) of the insect (Figure 5). This analysis revealed that a higher number of bacteria exhibited significant specificity to the insect organ (7 families) compared to the sex (1 family). At the family level, only Blattabacteriaceae (order Flavobacteriales) exhibited a strong specificity toward the insect sex (spec = −0.585, *p* = 0.041) (Figure 5a). Although members of this family were detected in both males and females, a greater abundance was found in females (up to 8.2-fold) (Figure 5b). Seven families revealed statistically significant specificity for insects’ organs (average spec = −0.355, *p* ≤ 0.047) (Figure 5a). Among these, Microbacteriaceae (order Micrococcales) exhibited the strongest specificity (spec = −0.676, *p* = 0.014), with higher abundance in the genitalia and almost absent in the abdomen (Figure 5b). The other organ-specific families were generally more abundant in either the head (Bacillaceae, Staphylococcaceae, Halomonadaceae, Moraxellaceae, and Sphingomonadaceae) or the genitalia (Propionibacteriaceae) (Figure 5b).

## 4. Discussion

In this work, we investigated the bacterial community inhabiting different organs of males and females of *P. spumarius*, using both culture-dependent and independent (metabarcoding) approaches. Results showed that only 8.9% of the genera found in the metabarcoding approach were similarly detected in the culture-dependent approach (Figure S1). This was an expected result due to the difficulties in culturing many endosymbiotic bacteria in axenic culture media [17]. Indeed, numerous endosymbionts inhabiting insects rely on their host and other microbial community members for metabolic functions and development [36]. To mimic the chemical composition of the insect host, we supplemented a primary medium (PCA) with crushed insects or commercial insect medium. However, such supplementation did not stimulate bacterial growth more than the primary medium, leading to similar results in diversity and abundance. The nutritional requirements for bacterial growth also explain why the well-represented genera identified through the culture-dependent method differed from those identified via metabarcoding. According to this, the genera *Clavibacter*, *Kineococcus*, *Mycoplasma*, *Patulibacter*, *Rathayibacter*, *Rhizobium*, and *Subtercola* obtained via culturing were not detected using metabarcoding (Figure S1). Thus, a combination of the culturing and metabarcoding methods appears to be the most suitable procedure to accurately characterize the *P. spumarius* bacterial community, as observed by other researchers studying different insect species [37,38]. Moreover, the culturing approach enables the isolation of bacterial strains, which can be further tested individually or in combination to decipher their ecological roles in *P. spumarius*. Indeed, this was the primary purpose of incorporating the culturing method into this work. In turn, the metabarcoding approach used allowed the detection of several bacterial genera (*Rhodococcus*, *Citrobacter*, *Halomonas*, *Streptomyces*, and *Providencia*) for the first time in *P. spumarius*, thereby broadening the spectrum of symbionts associated with this insect.

### 4.1. Prevalence of Candidatus Sulcia and Cutibacterium in P. spumarius Population

The whole bacteriome of *P. spumarius* assessed by the metabarcoding approach shows that the most predominant phyla identified as ASVs were Proteobacteria, followed by Bacteroidota and Actinobacteria. This result is consistent with previous studies conducted on *Philaenus* spp. [13] and other species, such as *Anoplophora chinensis* [37] and *Hermetia illucens* [38]. A similar picture was observed for the identified OTUs from bacterial isolates, with the phyla Actinobacteria and Proteobacteria being the most abundant. In *P. spumarius*, the most prevalent ASV bacterial genera were *Candidatus* Sulcia (Bacteroidota) and *Cutibacterium* (Actinobacteria), while the most frequently isolated taxa were from the genera *Curtobacterium* (Actinobacteria), *Pseudomonas* (Proteobacteria), and *Rhizobiaceae* sp1. (Proteobacteria). The genus *Candidatus* Sulcia encompasses members like *Candidatus* Sulcia muelleri, which are obligate endosymbionts associated with a wide range of sap-feeding hemipteran insects [39], including *P. spumarius* [13]. *Candidatus* Sulcia is known to inhabit specialized abdomen cells, called bacteriocytes, where it plays an important role by providing the hosts with essential amino acids [40]. Thus, the high abundance of *Candidatus* Sulcia in *P. spumarius*, particularly in the insects’ abdomen, suggests that this endosymbiont is essential for the hosts’ survival by supplying essential amino acids. This is particularly important for *P. spumarius*, which feeds exclusively on nutrition-poor xylem sap. The other prevalent genus (*Cutibacterium*) has been reported to dominate the gut of several insects, such as mosquitos [41], ants [42], and spiders [43], as well as the endophytic community of several plant species [44,45], including olive trees, where this bacterial genus was found inhabiting the xylem sap [46]. Altogether, this evidence suggests that *P. spumarius* may acquire *Cutibacterium* spp. bacteria by feeding on xylem sap. This is the first report of the *Cutibacterium* genus in *P. spumarius*, but its role in host insect fitness is a topic deserving of further study. 

Although *P. spumarius* could play a role in the spread of the pathogen *X. fastidiosa* (Xanthomonadaceae family), neither isolates nor sequences from this species were detected in the studied population of *P. spumarius*, which was sampled in north Portugal. However, ASVs from other genera of the same family, encompassing known plant pathogens (e.g., *Stenotrophomonas*, *Pseudoxanthomonas*, and *Thermomonas*), were detected in the surveyed *P. spumarius* adults, although in low abundance (less than 1% of the relative abundance of reads in the total dataset). Our study also reveals the presence of several bacterial genera in *P. spumarius* comprising important plant pathogens, such as *Pseudomonas* (both as bacterial isolates or ASVs) [47], *Curtobacterium* (as bacterial isolates) [48], and *Rathayibacter* (as bacterial isolates) [49]. These findings suggest the possibility that *P. spumarius* may also be a vector for these phytopathogens.

### 4.2. Host Organ and, to a Lesser Extent, Sex Shape the Diversity and Structure of the P. spumarius Bacterial Community

While culturing and metabarcoding methods offer complementary insights, only information derived from the culture-independent approach was used to explore variations in the diversity and structure of bacterial communities among different organs or sexes of *P. spumarius*. The results showed that the bacterial composition of *P. spumarius* is mainly affected by the organ compared to the sex. This is seen through several insect microbiome studies, such as those involving cicadas [50] and mosquitos [51], confirming that bacterial communities varied in an organ-specific manner. The bacterial composition of *P. spumarius* is also influenced by sex, although to a lesser extent when compared to organs. This difference among sexes is likely due to the physiological differences between males and females, as previously suggested for other insects [52]. However, earlier studies have reported contradictory results about the influence [5,6,52] or not [53,54] of sex on insect bacterial composition.

### 4.3. Microbacteriaceae Is Organ-Specific, While Blattabacteriaceae Is Sex-Specific

A set of bacterial families showed a high organ-specificity index. Thus, the specific presence of these bacteria in a particular organ might reflect the different functions these microorganisms play in that specific organ of *P. spumarius*. Otherwise, these bacteria would not be highly associated with each organ. These findings may also reflect organ-bacteria adaptations, similar to what was previously reported for other insects [50,51]. The bacterial family showing the strongest organ-specificity was Microbacteriaceae, which was more specific to genitalia. This family mainly encompasses the genus *Curtobacterium*, followed by the genera *Agrococcus*, *Amnibacterium*, *Frigoribacterium*, *Microbacterium*, and *Okibacterium* (Figure S1b). Curiously, *Curtobacterium* was one of the most frequent isolates obtained from females. However, the specific biological functions of these genera in insects have not yet been reported. Most members of these genera have been described as endophytes of several agronomic crops and wild plants, being reported to provide several beneficial effects on their plant host [55,56]. Except for *Agrococcus* spp., all the other bacterial genera were exclusively detected or found in a higher abundance in the genitalia of *P. spumarius*. Thus, we hypothesized that these bacteria might be relevant to the reproductive system of *P. spumarius*. Nevertheless, further investigation is needed to explore this possibility in detail.

Likewise, several bacterial families were uniquely detected in either males (16 families) or females (9 families) of *P. spumarius*. In particular, the Blattabacteriaceae family displayed a high specificity index for sex. These results may suggest different types of interactions between males and females and their respective bacterial communities, which could result in different outcomes for insect host performance, as reported previously for other insects [57]. Blattabacteriaceae encompasses the known obligate endosymbiont *Candidatus* Sulcia, found in greater abundance in females than males. This endosymbiont is transferred from mothers to offspring transovarially within female germ cells [58]. Thus, females seem to invest more in ensuring the successful transmission of *Candidatus* Sulcia to the next generation, resulting in a higher abundance of this endosymbiont in their bodies. 

### 4.4. Philaenus spumarius Harbours a High Diversity of Symbiotic Bacteria

The surveyed adults of *P. spumarius* showed that they harbored a number of ASVs from symbiotic bacteria, which could play important roles for the insect. Indeed, the identified bacteria belonged to genera that have been recognized to manipulate insect reproduction via male-killing and parthenogenesis (*Rickettsia* and *Flavobacterium*) [59,60], supply amino acids and vitamins to their hosts (*Enterobacter*, *Sodalis*, *Rhodococcus*, and *Pantoea*) [61,62,63,64], or provide insecticide resistance to their host (*Rickettsia* and *Citrobacter*) [65,66]. Some are also capable of fixing nitrogen within the insect host (*Enterobacter*) [37], producing antibiotics (*Streptomyces*) [67], and providing insect protection against parasitoids (e.g., *Serratia* and *Providencia*) [68,69] or heat stress (*Serratia* and *Sodalis*) [70,71]. The *Halomonas* genus was also detected in our study, but although members have been recognized as insect symbionts [50], their specific role has yet to be determined. Among the endosymbiotic genera identified, only *Rickettsia*, *Flavobacterium*, *Pantoea*, *Serratia*, and *Sodalis* [12,13,63] have been previously reported in *P. spumarius*. Interestingly, the common facultative endosymbiont *Wolbachia* spp., which had previously been identified in the Greek populations of *P. spumarius* [10,12], was not detected in the Portuguese population. However, in accordance with our study, both Formisano et al. [11] and Kolasa et al. [13] described the *Wolbachia* genus as being absent in Southern Europe and warmer climates. The rarity of this endosymbiont in southern *P. spumarius* populations is suggested to be related to environmental factors, including temperature and humidity [11]. Indeed, hot and dry climates may influence the prevalence and persistence of *Wolbachia* in insect populations by negatively affecting its transmission [11]. Notably, some of the symbiotic genera identified in our work were exclusively found in specific organs, mainly in genitalia (e.g., *Citrobacter*, *Rhodococcus*, and *Flavobacterium*) or in a particular sex, either in males (e.g., *Rickettsia*, *Citrobacter*, and *Rhodococcus*) or females (e.g., *Providencia*, *Streptomyces*, and *Flavobacterium*). This result reflects once more the bacterial adaptation to a specific organ or sex, where they are likely to play important biological roles in *P. spumarius*. A set of bacterial genera, whose biological function in insects is still unknown, was also detected in *P. spumarius* at high abundance, some of which have been reported in insects, such as the genus *Staphylococcus* [50,72].

## 5. Conclusions

Overall, the results reveal that natural populations of *P. spumarius* are colonized by a diverse bacterial community dominated by Proteobacteria, Bacteroidota, and Actinobacteria phyla. The genera *Candidatus* Sulcia and *Cutibacterium* were the most prevalent ASVs, and *Curtobacterium*, *Pseudomonas*, and *Rhizobiaceae* sp1. were the most frequently isolated taxa. This observation suggests that these genera might be vital for the host. The organs (head, abdomen, and genitalia) and, to a lesser extent, the sex of *P. spumarius* are factors that significantly structure the bacterial composition. Accordingly, some bacterial taxa displayed a high level of specificity to the organ (particularly the Microbacteriaceae family) or the sex (Blattabacteriaceae family). Members of Microbacteriaceae were most abundant in the genitalia, while members of Blattabacteriaceae dominated the female bacteriome. This exciting result may represent an adaptation and specificity of these bacterial families to particular organs or sexes, potentially implicating the fitness of the host, *P. spumarius*. In this work, we identified a set of symbiotic bacteria as ASV for the first time in *P. spumarius* (belonging to the genera *Rhodococcus*, *Citrobacter*, *Halomonas*, *Streptomyces*, and *Providencia*), expanding the endosymbiont repertoire of this insect. Moreover, several bacterial genera were found to dominate the bacteriome of *P. spumarius*, but their biological function in insects remains unknown. Taken together, the results of our study enhance our understanding of the relationship between *P. spumarius* and associated bacteria. However, further studies on the interaction between *P. spumarius* microorganisms are required to identify the functional role of these microorganisms and the outcomes of their interaction. The obtained information could provide new clues for designing vector control strategies relying on *P. spumarius*-associated bacteria.

## Data Availability

All 16S rRNA sequences generated in the present study were submitted to the NCBI database with the accession numbers OM243853 to OM243910. Similarly, the raw sequence reads and respective metadata from metabarcoding analysis were deposited in the NCBI Sequence Reads Archive (SRA) database under the BioProject ID PRJNA797278.

## Supplementary Materials

The following supporting information can be downloaded at https://www.mdpi.com/article/10.3390/microorganisms12020298/s1, Table S1: Locations and GPS coordinates of the sites where *Philaenus spumarius* adults were collected; Table S2: BLAST results showing the final taxonomic classification of the cultivable bacteria isolated from *Philaenus spumarius* adults; Table S3: Abundance (Log CFU/mL) of each bacterial genera detected in the different *Philaenus spumarius* organs (abdomen, head, and genitalia) and sex (females and males) using a culture-dependent approach; Table S4: Processing of sequences obtained from the metabarcoding approach via Illumina MiSeq sequencing of the V4 region of 16S rRNA gene; Table S5: Results from PERMANOVA and pairwise comparisons PERMANOVA (R^2^ and *p*-value) of the bacterial community composition between the different *Philaenus spumarius* organs (abdomen, head, and genitalia) and sex (females and males); Figure S1: Krona charts representing the relative abundance of the whole bacterial communities detected using culture-dependent (a) and culture-independent (b) approaches. Figures were constructed using Krona tool (Ondov et al., 2011 [73]); Figure S2: Relative read abundance of the symbiotic bacterial community (at genus level) in females and males of *Philaenus spumarius* in different organs (abdomen, head, and genitalia) and the other organs from both insect sexes.
