# Supplementary material for: Diversity, Composition, and Specificity of the Philaenus spumarius Bacteriome
Source: Microorganisms. 2024 Jan 30;12(2):298. doi: 10.3390/microorganisms12020298 (PMC10893442; doi:10.3390/microorganisms12020298)
Supplement: Supplementary file 1 [file microorganisms-12-00298-s001.zip › microorganisms-2827902-supplementary.pdf]

Supplementary Material

Table S1: Locations and GPS coordinates of the sites where *Philaenus spumarius* adults were collected.

Location name	GPS coordinates
Caracoi	41º18’46.1’’N 8º38’10.4’’W
Cabeceiras de Basto	41º29’56.6’’N 8º00’24.2’’W
Bragança	41º48’33’’N 6º32’48’’W
Pinelo	41º38’38’’N 6º32’48’’W

Table S2: BLAST results showing the final taxonomic classification of the cultivable bacteria isolated from *Philaenus spumarius* adults.

Final classification (accession number)	Query cover (%)	*E value*	Percentage of identity (%)	BLAST classification (accession number)
Williamsia phyllosphaerae (OM243910.1)	100	0.0	99.54	*Williamsia phyllosphaerae* (MG205541.1)
Rathayibacter festucae (OM243909.1)	100	0.0	99.33	*Rathayibacter festucae* (LN774180.1)
Rhizobiaceae (OM243908.1)	100	0.0	96.92	*Rhizobium* (OK614003.1)
Curtobacterium herbarum (OM243907.1)	100	0.0	100	*Curtobacterium herbarum* (MH298411.1)
Aeromicrobium (OM243906.1)	99	0.0	100	*Aeromicrobium* (JX949248.1)
Aeromicrobium (OM243905.1)	100	0.0	100	*Aeromicrobium* (KP639161.1)
Clavibacter michiganensis (OM243904.1)	100	0.0	97.26	*Clavibacter* (MH813366.1)
Frigoribacterium (OM243903.1)	100	0.0	100	*Frigoribacterium* (KF551104.1)
Erwiniaceae (OM243902.1)	100	0.0	99.77	*Pantoea agglomerans* (MT270698.1)
Erwiniaceae (OM243901.1)	100	0.0	99.79	*Erwinia* (MK070123.1)
Pseudomonas graminis (OM243900.1)	100	0.0	99.32	*Pseudomonas graminis* (CP053746.1)
Methylobacterium cerastii (OM243899.1)	99	0.0	99.03	*Methylobacterium* (FN868947.1)
Rhizobiaceae (OM243898.1)	100	0.0	100	*Rhizobium nepotum* (MN826216.1)
Brevundimonas (OM243897.1)	100	0.0	99.76	*Brevundimonas intermedia* (OK135613.1)
Staphylococcus xylosus (OM243896.1)	100	0.0	100	*Staphylococcus xylosus* (MN758801.1)
Pseudomonas (OM243895.1)	100	0.0	99.56	*Pseudomonas lutea* (MH298412.1)
Methylobacterium (OM243894.1)	100	0.0	100	*Methylobacterium* (MT360249.1)
Rhizobium (OM243893.1)	100	0.0	99.75	*Rhizobium selenitireducens* (MH843496.1)
Bacillus (OM243892.1)	100	0.0	100	*Bacillus cereus* (OP559473.1)
Rhizobiaceae (OM243891.1)	100	0.0	100	*Rhizobium nepotum* (MN826216.1)
Kineococcus (OM243890.1)	99	0.0	98.38	Uncultured *Kineococcus* (KF508555.1)
Aeromicrobium fastidiosum (OM243889.1)	100	0.0	99.77	*Aeromicrobium fastidiosum* (KM434774.1)
Pseudomonas (OM243888.1)	100	0.0	99.78	*Pseudomonas brassicacearum* (KJ438148.1)
Pseudomonas (OM243887.1)	100	0.0	100	*Pseudomonas brassicacearum* (KJ438148.1)

Rhizobiaceae (OM243886.1)	100	0.0	100	*Rhizobium nepotum* (MN826216.1)
Subtercola (OM243885.1)	100	0.0	98.20	*Subtercola* (HQ256860.1)
Aeromicrobium (OM243884.1)	99	0.0	100	*Aeromicrobium* (JX949248.1)
Pseudomonas (OM243883.1)	99	0.0	100	*Pseudomonas* (KY678889.1)
Pseudomonas (OM243882.1)	100	0.0	99.57	*Pseudomonas* (MW496941.1)
Rhizobiaceae (OM243881.1)	100	0.0	100	*Rhizobium nepotum* (MN826216.1)
Rathayibacter festucae (OM243880.1)	100	0.0	100	*Rathayibacter* (MN811053.1)
Aeromicrobium (OM243879.1)	100	0.0	100	*Aeromicrobium* (KP195230.1)
Rhodococcus (OM243878.1)	100	0.0	100	*Rhodococcus cerastii* (ON056038.1)
Williamsia maris (OM243877.1)	100	0.0	99.77	*Williamsia maris* (NR_024671.1)
Clavibacter michiganensis (OM243876.1)	100	0.0	100	*Clavibacter michiganensis* (KR922179.1)
Williamsia phyllosphaerae (OM243875.1)	100	0.0	99.54	*Williamsia phyllosphaerae* (MG205541.1)
Methylobacterium adhaesivum (OM243874.1)	100	0.0	99.76	*Methylobacterium adhaesivum* (MK138652.1)
Pseudomonas (OM243873.1)	100	0.0	100	*Pseudomonas* (KY678889.1)
Rhodococcus fascians (OM243872.1)	100	0.0	99.77	*Rhodococcus* (MN366368.1)
Frigoribacterium faeni (OM243871.1)	100	0.0	99.55	*Frigoribacterium faeni* (LN774201.1)
Sphingomonas (OM243870.1)	100	0.0	100	*Sphingomonas* (KR922270.1)
Chryseobacterium (OM243869.1)	100	0.0	99.34	*Chryseobacterium psychrotolerans* (LR215140.1)
Rhodococcus corynebacterioides (OM243868.1)	100	0.0	100	*Rhodococcus corynebacterioides* (MW712715.1)
Subtercola (OM243867.1)	100	0.0	97.99	*Subtercola* (KF551101.1)
Aeromicrobium (OM243866.1)	100	0.0	99.77	*Aeromicrobium kwangyangensis* (EU834249.1)
Microbacterium (OM243865.1)	99	0.0	100	*Microbacterium* (MH985312.1)
Pseudomonas (OM243864.1)	100	0.0	100	*Pseudomonas brassicacearum* (KJ438148.1)
Patulibacter minatonensis (OM243863.1)	100	0.0	99.56	*Patulibacter* (KJ654735.1)
Bacillus (OM243862.1)	100	0.0	100	*Bacillus cereus* (OP559473.1)
Methylobacterium (OM243861.1)	99	0.0	100	*Methylobacterium* (MT360209.1)

Staphylococcus xylosus (OM243860.1)	100	0.0	100	*Staphylococcus xylosus* (MN758801.1)
Curtobacterium flaccumfaciens (OM243859.1)	100	0.0	100	*Curtobacterium flaccumfaciens* (MT323132.1)
Aeromicrobium (OM243858.1)	100	0.0	100	*Aeromicrobium* (MH512420.1)
Sphingomonas (OM243857.1)	100	0.0	100	*Sphingomonas* (MN989153.1)
Rathayibacter festucae (OM243856.1)	100	0.0	100	*Rathayibacter festucae* (MT431571.1)
Rhizobiaceae (OM243855.1)	100	0.0	100	*Rhizobium nepotum* (MN826216.1)
Aeromicrobium (OM243854.1)	100	0.0	99.77	*Aeromicrobium* (MH512420.1)
Rhodococcus corynebacterioides (OM243853.1)	100	0.0	99.77	*Rhodococcus* (MN811083.1)

Table S3: Abundance (Log CFU/mL) of each bacterial genera detected in the different *Philaenus spumarius* organs (abdomen, head, and genitalia) and sex (females and males), by culture-dependent approach.

Bacterial genus/family	Female			Male			Total				
	Abdomen	Head	Genitalia	Abdomen	Head	Genitalia	Abdomen	Head	Genitalia	Female	Male
Aeromicrobium	4.05	0	4.56	0	0	0	4.05	0	4.56	4.68	0
Bacillus	0	0	0	0	0	0	0	0	0	0	0
Brevundimonas	0	3.00	0	2.52	0	0	2.52	3.00	0	3.00	2.52
Chryseobacterium	0	0	0	0	0	0	0	0	0	0	0
Clavibacter	0	0	3.64	3.52	0	0	3.52	0	3.64	3.64	3.52
Curtobacterium	5.85	3.22	0	0	0	0	5.85	3.22	0	5.85	0
Erwiniaceae	2.52	0	2.82	0	0	0	2.52	0	2.82	3.00	0
Frigoribacterium	0	0	3.64	0	0	0	0	0	3.64	3.64	0
Kineococcus	0	0	2.52	0	0	0	0	0	2.52	2.52	0
Methylobacterium	3.30	0	2.82	0	0	0	3.30	0	2.82	3.43	0
Microbacterium	0	0	0	0	0	0	0	0	0	0	0
Mycoplasma	0	0	3.64	3.00	0	0	3.00	0	3.64	3.64	3.00
Patulibacter	3.12	0	0	0	0	0	3.12	0	0	3.12	0
Pseudomonas	0	3.12	4.31	0	0	5.08	0	3.12	5.14	4.34	5.08
Rathayibacter	3.43	0	2.82	4.01	0	0	4.11	0	2.82	3.52	4.01
Rhizobiaceae	0	5.27	3.00	0	0	0	0	5.27	3.00	5.27	0
Rhizobium	0	0	0	3.12	2.52	0	3.12	2.52	0	0	3.22
Rhodococcus	4.30	0	3.88	0	0	3.94	4.30	0	4.21	4.44	3.94
Staphylococcus	5.10	2.52	3.94	0	0	0	5.10	2.52	3.94	5.13	0
Subtercola	3.22	0	0	0	0	0	3.22	0	0	3.22	0
Williamsia	3.85	0	3.37	0	0	2.52	3.85	0	3.43	3.97	2.52

Table S4: Processing of sequences obtained from the metabarcoding approach via *Illumina MiSeq* sequencing of the V4 region of 16S rRNA gene.

**Sample**			**Raw reads**	**Merged reads**	**Processed reads**	**Classified reads into ASVs**
**Female**	Abdomen	FA1	11,195	10,762	10,754	10,651
		FA2	15,483	15,005	15,003	14,915
		FA3	11,816	11,367	11,362	11,253
		FA4	12,645	12,227	12,101	11,960
		FA5	8,906	8,641	8,640	8,601
	Head	FC1	26,625	25,704	25,684	24,643
		FC2	26,158	25,475	25,467	25,009
		FC3	21,012	20,462	20,458	19,863
		FC4	20,909	20,358	20,351	20,191
		FC5	60,779	59,047	59,040	56,360
	Genitalia	FG1	39,602	37,410	37,395	35,797
		FG2	35,293	33,808	33,757	32,074
		FG3	28,249	26,724	26,707	25,954
		FG4	22,012	21,299	21,295	20,659
		FG5	20,790	19,551	19,544	19,132
**Male**	Abdomen	MA1	39,835	38,699	38,698	38,180
		MA2	31,254	30,252	30,245	17,741
		MA3	40,593	39,329	39,326	37,272
		MA4	32,645	30,062	29,978	29,313
		MA5	38,987	37,849	37,843	36,285
	Head	MC1	22,974	22,330	22,328	21,957
		MC2	27,851	27,109	27,106	25,331
		MC3	15,966	15,522	15,519	14,883
		MC4	23,864	23,022	23,012	22,112
		MC5	20,693	20,108	20,103	19,455
	Genitalia	MG1	17,502	16,492	16,487	15,957
		MG2	30,169	28,521	28,508	24,068
		MG3	27,838	26,430	26,421	21,159
		MG4	21,025	19,791	19,783	19,088
		MG5	24,878	23,599	23,555	22,523
**Total**			777,548	746,955	746,470	702,386

Table S5: Results from PERMANOVA and pairwise comparisons PERMANOVA (R^2 and *p*-value) of the bacterial community composition between the different *Philaenus spumarius* organs (abdomen, head, and genitalia) and sex (females and males).

	R^2	*p-value*
Organ	0.2595	0.001
Sex	0.1364	0.001
Organ × sex (interaction)	0.0817	0.035
Abdomen vs head	0.1552	0.015
Abdomen vs genitalia	0.2960	0.001
Head vs genitalia	0.1645	0.001
♀ *head vs* ♂ *head*	0.3313	0.016
♀ *abdomen vs* ♂ *abdomen*	0.3730	0.016
♀ *genitalia vs* ♂ *genitalia*	0.1602	0.016

a

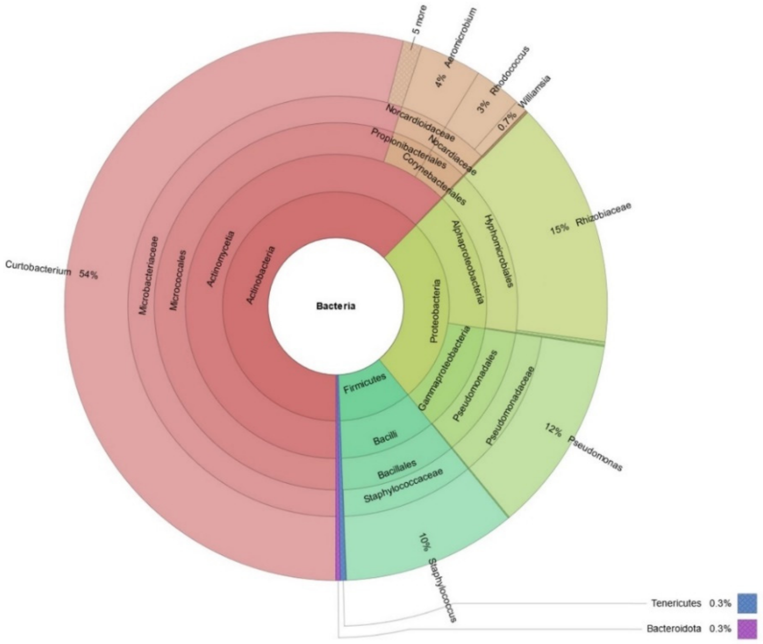

b

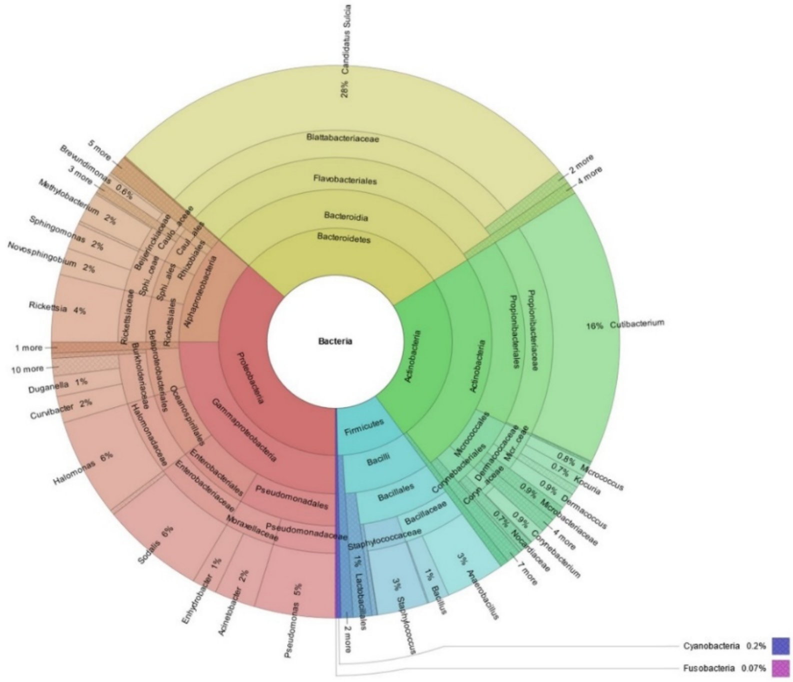

Figure S1: Krona charts representing the relative abundance of the whole bacterial communities detected by culture-dependent (**a**) and culture-independent **(b**) approaches. Figures were constructed using Krona tool [73].

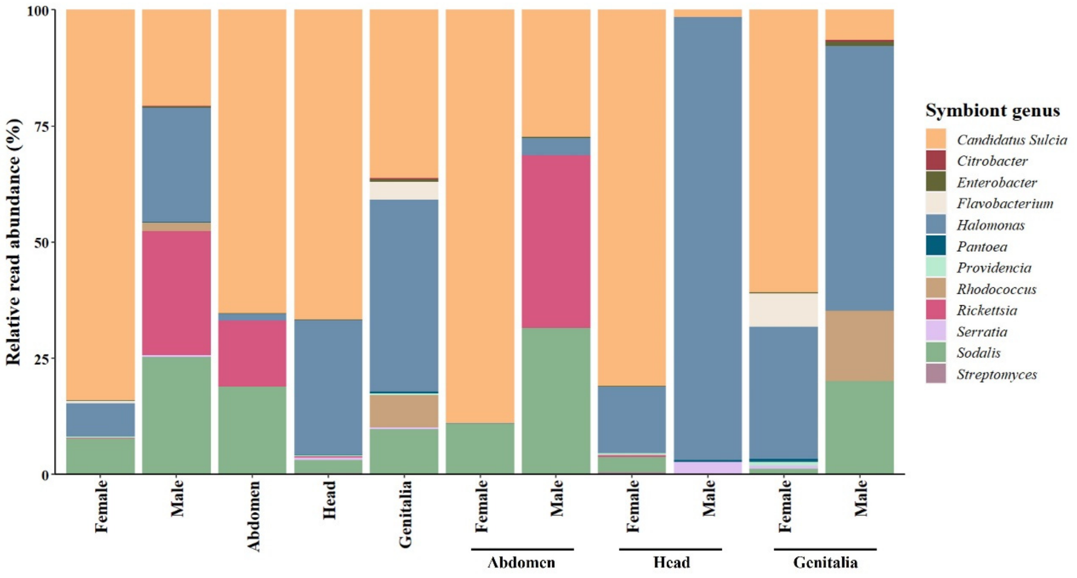

Figure S2: Relative read abundance of the symbiotic bacterial community (at genus level) in females and males of *Philaenus spumarius* in different organs (abdomen, head, and genitalia) and the other organs from both insect sexes.
